# Ascending aorta curvature and flow displacement are associated with accelerated aortic growth at long-term follow-up: A MRI study in Marfan and thoracic aortic aneurysm patients

**DOI:** 10.1016/j.ijcha.2021.100926

**Published:** 2021-12-13

**Authors:** M.J.P. van Hout, J.F. Juffermans, H.J. Lamb, E.S.J. Kröner, P.J. van den Boogaard, M.J. Schalij, I.A. Dekkers, A.J. Scholte, J.J. Westenberg

**Affiliations:** a Department of Cardiology, Leiden University Medical Center, Albinusdreef 2, 2333 ZA Leiden, the Netherlands; b Department of Radiology, Leiden University Medical Center, Albinusdreef 2, 2333 ZA Leiden, the Netherlands

**Keywords:** Marfan syndrome, Thoracic aortic aneurysm, Ascending aorta curvature radius, Flow displacement, Long-term follow-up

## Abstract

**Background:**

Aortic aneurysm formation is associated with increased risk of aortic dissection. Current diagnostic strategies are focused on diameter growth, the predictive value of aortic morphology and function remains underinvestigated. We aimed to assess the long-term prognostic value of ascending aorta (AA) curvature radius, regional pulse wave velocity (PWV) and flow displacement (FD) on aortic dilatation/elongation and evaluated adverse outcomes (proximal aortic surgery, dissection/rupture, death) in Marfan and non-syndromic thoracic aortic aneurysm (NTAA) patients.

**Methods:**

Long-term magnetic resonance imaging (MRI) and clinical follow-up of two previous studies consisting of 21 Marfan and 40 NTAA patients were collected. Baseline regional PWV, AA curvature radius and normalized FD were assessed as well as diameter and length growth rate at follow-up. Multivariate linear regression was performed to evaluate whether baseline predictors were associated with aortic growth.=.

**Results:**

Of the 61 patients, 49 patients were included with MRI follow-up (n = 44) and/or adverse aortic events (n = 7). Six had undergone aortic surgery, no dissection/rupture occurred and one patient died during follow-up. During 8.0 [7.3–10.7] years of follow-up, AA growth rate was 0.40 ± 0.31 mm/year. After correction for confounders, AA curvature radius (p = 0.01), but not FD or PWV, was a predictor of AA dilatation. Only FD was associated with AA elongation (p = 0.01).

**Conclusion:**

In Marfan and non-syndromic thoracic aortic aneurysm patients, ascending aorta curvature radius and flow displacement are associated with accelerated aortic growth at long-term follow-up. These markers may aid in the risk stratification of ascending aorta elongation and aneurysm formation.

## 1 Introduction

Thoracic aortic aneurysms are associated with increased risk of dissection and rupture which are potentially fatal events, therefore close monitoring of patients with aortic aneurysms is crucial [1]. Patients with Marfan syndrome are at increased risk of aortic aneurysm formation, particularly of the aortic root and ascending aorta. However, considering approximately 5% of aortic aneurysms are related to Marfan syndrome, most thoracic aneurysm are found in other syndromes and non-syndromic patients [2]. Up to now, international guidelines have focused on aortic diameter for risk assessment of dissection and guidance for pre-emptive surgery, while aortic length has been underrecognized as an important morphological parameter [3]. Assessment of aortic diameter alone has shown to be insufficient, as >50% of aortic dissections occur before the intervention threshold is reached [4]. Several studies have demonstrated that higher levels of aortic elongation are associated with increased risk of aortic dissection [5], [6]. Magnetic resonance imaging (MRI) is capable of providing morphological as well as functional information such as flow displacement and arterial stiffness assessed through pulse wave velocity (PWV) and may improve risk stratification of aortic aneurysms in high risk patients. MRI allows for PWV assessment locally in the aorta through multi-slice 2D imaging in oblique-sagittal ‘candy-cane’ view with in-plane velocity-encoding [7]. Local PWV enables assessment of wall stiffness near the area at risk, which may be relevant for aneurysm prediction as aneurysm formation is often regional. Normal regional PWV is associated with absence of increased aortic diameter and is able to predict absence of regional aortic diameter growth in Marfan patients at 2-year follow-up [7], [8]. Besides arterial stiffness, other functional and morphological parameters have been linked to aortic remodeling. In a mathematical model, ascending aorta curvature has shown to be an important factor in the amount of force that is exerted on the vessel wall and thereby contributes to aneurysm formation and risk of dissection [9]. Furthermore, flow displacement in the ascending aorta, a marker of flow eccentricity relative to the aortic lumen centerline, has been associated with aortic dilatation in bicuspid valve patients [10]. However, to date little is known about the impact of flow displacement on aortic growth at follow-up in other patient populations. Therefore, in the current study we aimed to assess the long-term prognostic value of regional PWV, ascending aorta curvature radius and flow displacement on aortic diameter and length growth rate in Marfan and non-syndromic thoracic aortic aneurysm patients.

## 2 Methods

### 2.1 Patient population

We performed a combined analysis of two previous studies, in which 21 Marfan patients and 40 non-syndromic thoracic aortic aneurysm (NTAA) patients were included who were followed regularly at the outpatient clinic of the cardiology department of the Leiden University Medical Center, and who received MRI including in-plane PWV assessment of the aorta at baseline [7], [8]. In these patients long-term follow-up including MRI and adverse aortic events (proximal aortic surgery, aortic dissection/rupture or death) were assessed. Follow-up imaging was performed as part of routine clinical care. The latest scan was used for follow-up analysis with a minimum of 4 years between the first and the follow-up scan. Patient medical records were checked to see whether patients had endured an aortic dissection or rupture, undergone aortic surgery (pre-emptive or acute) or had died of any cause up to 01-01-2021. For patients lost to follow-up, we checked the national records to verify if the patients were alive up to 01-01-2021. The ethics committee of the Leiden University Medical Center approved the study and waived the need for individual consent.

### 2.2 MRI acquisition at baseline

MRI at baseline was performed on a 1.5 T scanner (Philips Intera; Philips Healthcare, Best, the Netherlands) between March 2008 and December 2011. Imaging details have been previously described [7], [8], [11]. In short, contrast-enhanced MRA of the entire aorta was obtained from first-pass imaging of a 25 mL contrast bolus Dotarem (Guerbet, Gorinchem, the Netherlands), using a T1-weighted fast gradient-echo sequence during end-expiration breath-hold (85% rectangular field of view (FOV) 500 × 80 mm2, 50 slices of 1.6 mm slice thickness, echo time (TE) 1.3 ms, repetition time (TR) 4.6 ms, flip angle α 40°, acquisition voxel size 1.25 × 2.46 × 3.20 mm3). Regional PWV was determined from two consecutively acquired multi-slice 2D phase-contrast scans, positioned in oblique-sagittal orientation capturing the aorta in candy-cane view, with one-directional velocity-encoding respectively in phase-encoding (i.e., anterior-posterior) direction and in frequency-encoding (i.e., feet-head) direction. The velocity-sensitivity was set to 150 cm/s. Retrospective gating was performed with maximal number of phases reconstructed. The true temporal resolution was 8.6 ms (=2 × TR). Detailed scan parameters can be found in the previous studies [7], [8], [11].

### 2.3 MRI acquisition at follow-up

MRI was performed on a 3.0 T scanner (86% of the scans; Philips Ingenia, Philips Healthcare, Best, the Netherlands) or on a 1.5 T scanner (14% of the scans; Philips Ingenia, Philips Healthcare, Best, the Netherlands) on average 8.5 ± 2.0 years after baseline between 2015 and 2020. The thoracic aorta was imaged with a non-contrast-enhanced late-diastole Dixon MRI sequence with respiratory gating at expiration using a hemidiaphragm navigator (FOV 320 × 300 × 90 mm^3^, α 20°, TE_1_ 1.19, TE_2_ 2.37 ms, TR 3.7 ms, acquisition voxel size 0.7 × 0.7 × 1.8 mm^3^). Starting from 2018, the abdominal aorta was also imaged by two additional non-contrast-enhanced non-trigged Dixon MRI sequences during end-expiration breath-holds (FOV 480 × 300 × 240 mm^3^, α 10°, TE_1_ 1.19, TE_2_ 2.37 ms, TR 3.7 ms, acquisition voxel size 0.9 × 0.9 × 4 mm^3^; scanned in 64% of the population).

### 2.4 Image analysis

**Fig. 1:**
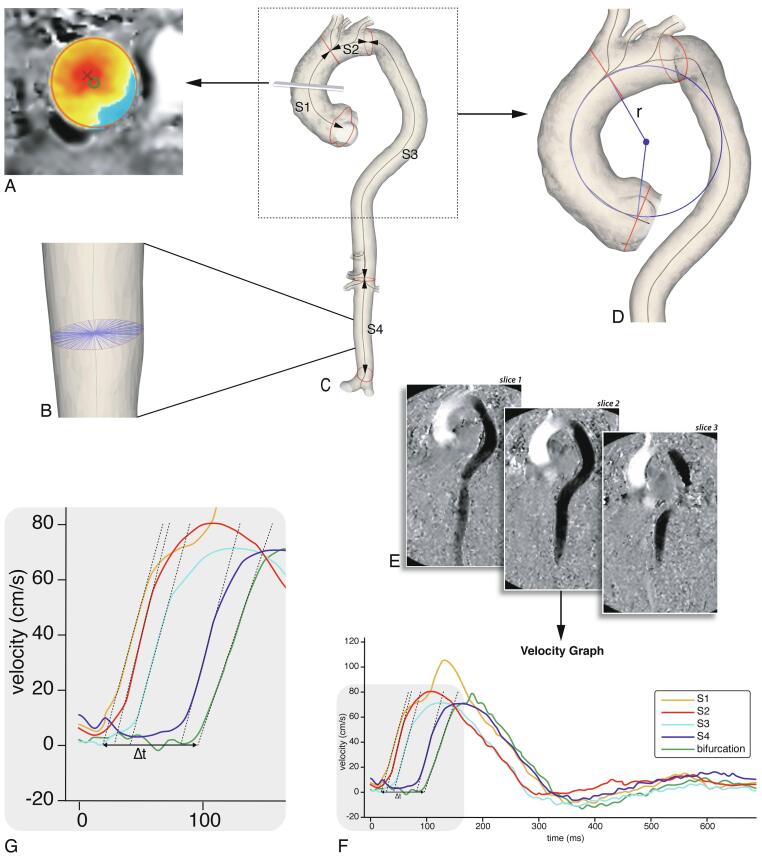
Imaging analysis. A: Flow displacement: the distance between the geometric lumen center (green circle) and the ‘center of forward flow velocity’ at peak systole (red×), normalized to lumen diameter. B: Aortic diameter: this was determined using radial spikes by first constructing a cross-section perpendicular to the centerline at every millimeter (one cross-section shown). At each cross-section the mean radial spike length was calculated and the largest mean diameter per segment was used. C: Baseline MRI 3D segmentation. The aorta was divided into four segments: ascending aorta (S1), aortic arch (S2), suprarenal descending aorta (S3) and infrarenal abdominal aorta (S4). Centerline length, maximal diameter and curvature radius were automatically calculated. D: Ascending aortic curvature radius: derived by fitting a circle through the 3D segmentation centerline, the radius (r) of the circle was used as a measure for ascending aorta curvature. E: PWV analysis using multi-slice in-plane velocity-encoded images (example shows feet-head direction). 200 sampling chords equally distributed along the aorta were automatically placed. For each chord the maximal velocity wave form was determined (F, G). Regional PWV was determined based on automated arrival time detection of each wave form in each segment. (For interpretation of the references to colour in this figure legend, the reader is referred to the web version of this article.)

MRI analysis was performed blinded to the patient characteristics. Maximum aortic diameter and length per segment were assessed at baseline and follow-up to calculate aortic growth rates. Additionally, at baseline regional PWV, ascending aorta curvature radius and ascending aorta normalized flow displacement were also assessed to subsequently test the predictive value of these markers on aortic diameter and length growth at long-term follow-up. Assessment of the different parameters are detailed below, an overview of these measures is provided in Fig. 1.

### 2.5 Aortic dimensions

Aortic lumen segmentation of all MRI images was performed on a Vitrea workstation (version 7.12, Vital Images Inc., Minnetonka, USA). Centerline length, maximal diameter and curvature radius were automatically calculated using in-house developed software after manually partitioning the aorta lumen into four longitudinal segments [12]. The four aortic segments were defined as follows: ascending aorta (S1), aortic arch (S2), suprarenal descending aorta (S3) and infrarenal abdominal aorta (S4) (Fig. 1c). The maximal aortic diameter was determined by first constructing a cross-section perpendicular to the centerline at every millimeter and fitting radial spikes across the diameter of the vessel through the centerline (Fig. 1b). Next, at each cross-section the mean radial spike length was calculated and the largest mean diameter per segment was used.

### 2.6 Ascending aorta curvature radius

The ascending aorta curvature radius was derived by fitting a circle through the segments’ centerline (Fig. 1d) [12]. The radius of the fitted circle was used as a measure for the curvature of the ascending aorta, in which a smaller radius corresponds to a more acutely angled aorta.

### 2.7 Regional pulse wave velocity

Regional PWV was calculated from the in-plane velocity-encoded data. The lumen of the aorta was manually segmented and aortic centerline was automatically detected. 200 sampling chords equally distributed along the aorta were automatically placed. For each chord the maximal velocity wave form was determined [7], [8], [11]. Regional PWV was determined based on automated arrival time detection of each wave form in each segment (Fig. 1e**-g**).

### 2.8 Normalized flow displacement

Normalized flow displacement is a measure of flow eccentricity and is defined as the distance between the geometric lumen center and the ‘center of forward flow velocity’ at peak systole, normalized to lumen diameter (Fig. 1a) [13]. Normalized flow displacement at baseline was assessed using CAAS MR Solutions 5.2 (Pie Medical Imaging, Maastricht, the Netherlands), based on 2D through-plane phase-contrast acquisitions of the ascending aorta at the level of the pulmonary trunk.

### 2.9 Statistical analysis

A complete case analysis was performed. Continuous variables were expressed as mean ± standard deviation (SD) or median [interquartile range] and nominal variables as number with corresponding percentage (%). Differences in means between Marfan and NTAA patients were compared using unpaired t-tests. The associations of arterial stiffness, ascending aorta curvature radius and ascending aorta flow displacement with regional aortic growth rate (diameter and length) were assessed using univariable and multivariable linear regression. Before multivariate regression, we evaluated the unadjusted association of potential covariates with both length and diameter growth separately. The following covariates we tested based on literature research: age, sex, baseline aortic diameter for association with diameter growth and baseline segment length for association with length growth, mean arterial pressure, body surface area, heart rate, smoking, Marfan syndrome, betablocker/angiotensin-converting enzyme (ACE)-inhibitor/angiotensin-II receptor blocker (ARB) use and history of diabetes or hypertension [14], [15], [16], [17], [18]. Covariates that were associated with ascending length or diameter growth with p < 0.20 were added to the corresponding multivariable analysis. Given the epidemiological as well as clinical relevance, sex and age were added to all models regardless of the association in univariable analysis. In the sensitivity analysis we also included ascending aortic diameters measured using transthoracic echocardiography (TTE) or computed tomography (CT) in clinical routine setting. A p-value of < 0.05 was considered significant. Analyses were performed using SPSS version 25.0 (SPSS Inc., Chicago, IL).

## 3 Results

**Fig. 2:**
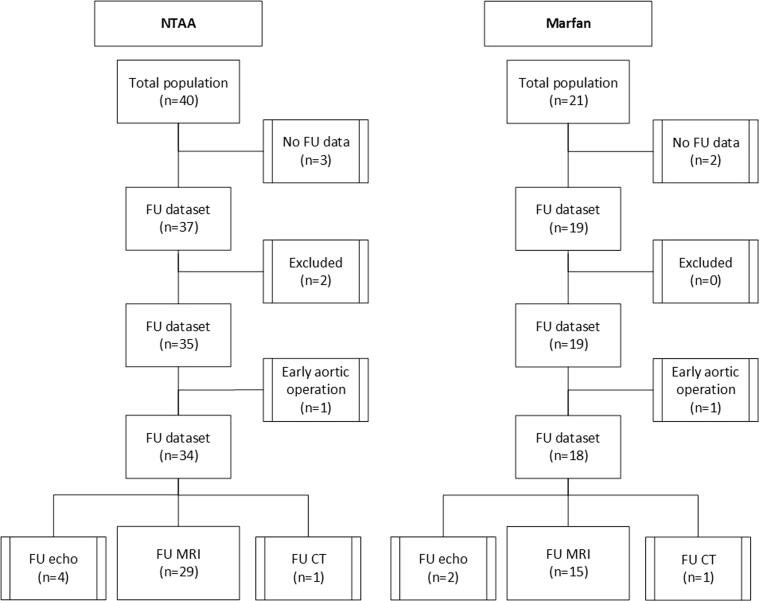
Flow chart. In total two patients were excluded, one due to absence of contrast-enhanced MRA images at baseline and one due to a type B dissection at baseline. Abbreviations: CT = computed tomography, FU = follow-up, MRI = magnetic resonance imaging, NTAA = non-syndromic thoracic aortic aneurysm.

Of the 61 patients, 44 patients had follow-up with MRI, two underwent aortic surgery < 4 years after the first scan, 7 were followed with TTE, 2 with CT and 5 were lost to follow-up. One NTAA patient who had TTE as follow-up imaging did not have contrast-enhanced MRA images at baseline and was therefore excluded (Fig. 2). Baseline characteristics of the population with MRI follow-up and/or adverse events are shown in Table 1. Mean age at baseline of the Marfan patients was 35 ± 13 years and the NTAA patients were 55 ± 14 years, 65% of the population was male. In correspondence with higher age, the NTAA patients had higher blood pressures, larger baseline diameters and length. When adjusted for age these differences between groups were not statistically significant. Baseline characteristics including patients with TTE and CT follow-up (which are used for sensitivity analysis) are shown in **Table S1** and demonstrate comparable characteristics.

**Table 1:** Baseline characteristics.

	**NTAA** *n = 31*	**Marfan** *n = 18*	**Total** *n = 49*
Age (years)	55.2 ± 13.6	34.7 ± 13.1	47.7 ± 16.6
Sex (% male)	23 (74.2%)	9 (50.0%)	32 (65.3%)
Height (cm)	180.3 ± 9.1	186.9 ± 9.5	182.8 ± 9.7
Weight (kg)	86.7 ± 13.5	84.9 ± 20.7	86.0 ± 16.3
BSA (m^2^)	2.1 ± 0.2	2.1 0.3	2.1 ± 0.2
SBP (mmHg)	135.6 ± 19.8	123.1 ± 11.3	130.8 ± 18.0
DBP (mmHg)	80.2 ± 10.6	72.4 ± 8.9	77.2 ± 10.6
MAP (mmHg)	98.7 ± 11.5	89.3 ± 8.5	95.1 ± 11.3
Heart rate (beats/min)	67.7 ± 9.6	66.2 ± 10.3	67.1 ± 9.8
History of - Hypertension (%) - Dyslipidaemia (%) - Diabetes (%) - Smoking (%) - Bicuspid aortic valve (%)	19 (61.3%) 10 (32.3%) 1 (3.2%) 5 (16.1%) 0 (0.0%)	2 (11.1%) 1 (5.6%) 0 (0%) 3 (16.7%) 0 (0.0%)	21 (42.9%) 11 (22.4%) 1 (2%) 8 (16.3%) 0 (0.0%)
Use of - AT2 / ACE inhibitor (%) - Betablocker (%) - Statin (%)	15 (48.4%) 14 (45.2%) 9 (29.0%)	5 (27.8%) 14 (77.8%) 0 (0.0%)	20 (40.8%) 28 (57.1%) 9 (18.4%)
Left ventricular ejection fraction	61.1 ± 5.2	61.1 ± 6.5	61.1 ± 5.7
Baseline diameter S1 (mm)	41.9 ± 4.1	38.4 ± 3.8	40.6 ± 4.3
Baseline diameter S2 (mm)	33.9 ± 3.7	26.9 ± 2.7	31.3 ± 4.8
Baseline diameter S3 (mm)	27.8 ± 2.6	24.7 ± 3.5	26.6 ± 3.3
Baseline diameter S4 (mm)	21.5 ± 2.5	18.9 ± 2.9	20.5 ± 2.9

Baseline length S1 (mm)	93.8 ± 13.0	83.4 ± 11.6	89.9 ± 13.4
Baseline length S2 (mm)	37.6 ± 7.4	32.9 ± 3.9	35.9 ± 6.6
Baseline length S3 (mm)	272.2 ± 24.2	253.1 ± 23.9	265.0 ± 25.6
Baseline length S4 (mm)	101.3 ± 10.6	104.0 ± 12.7	102.4 ± 11.4

PWV S1 (m/s)	7.4 ± 3.8	6.5 ± 6.0	7.1 ± 4.7
PWV S2 (m/s)	7.4 ± 5.0	6.0 ± 5.2	6.9 ± 5.1
PWV S3 (m/s)	8.1 ± 3.6	6.8 ± 4.5	7.6 ± 3.9
PWV S4 (m/s)	8.1 ± 4.3	8.0 ± 3.9	8.1 ± 4.1

Flow displacement	0.05 ± 0.04	0.04 ± 0.01	0.05 ± 0.04
Ascending aorta curvature radius (mm)	46.6 ± 7.5	44.6 ± 5.8	45.9 ± 6.9

*Data are shown as n (%), mean ± SD. Abbreviations: BSA = body surface area, DBP = diastolic blood pressure, MAP = mean arterial pressure, NTAA = non-syndromic thoracic aortic aneurysm, PWV = pulse wave velocity, SBP = systolic blood pressure.*			

### 3.1 Aortic growth and outcome during follow-up

Median time between follow-up scans was 8.0 [7.3–10.7] years. Mean aortic diameter growth rate of the ascending aorta (S1) was 0.40 ± 0.31 mm/year and was similar in Marfan and NTAA patients (Table 2). During this same period the ascending aortic length grew on average 0.98 ± 0.83 mm/year with again similar growth rates in Marfan and NTAA patients. Median follow-up time for adverse aortic events was 9.6 [9.3–11.8] years. Seven patients were operated, five received a root/ascending replacement, one received a thoracic endovascular aortic repair (TEVAR) for a known type B dissection and one received a Personalized External Aortic Root Support (PEARS). The NTAA patient with type-B dissection at baseline was excluded from further analysis. Of the patients lost to follow-up one NTAA patient died of unknown cause in 2020, all other patients were alive up to January 1, 2021. No new dissections occurred during follow-up.

**Table 2:** Average diameter and length growth.

	Diameter growth / year (mm/y)		Length growth / year (mm/y)	
	*NTAA*	*Marfan*	*NTAA*	*Marfan*
Aortic segment	**Mean** ± **SD**	**Mean** ± **SD**	**Mean** ± **SD**	**Mean** ± **SD**
S1	0.39 ± 0.33	0.42 ± 0.27	0.92 ± 0.94	1.09 ± 0.65
S2	0.51 ± 0.23	0.30 ± 0.20	0.58 ± 0.63	0.38 ± 0.55
S3	0.40 ± 0.21	0.31 ± 0.14	1.69 ± 1.27	1.37 ± 0.8
S4	0.14 ± 0.42	0.24 ± 0.28	0.90 ± 1.11	0.80 ± 0.75

Total			4.00 ± 1.30	3.54 ± 0.88

Abbreviations: NTAA = non-syndromic thoracic aortic aneurysm.

### 3.2 Pulse wave velocity, aortic curvature radius and flow displacement

At univariate analysis, age, baseline diameter and heart rate were associated with thoracic aortic diameter growth rate. Age, baseline length, history of hypertension and diabetes were associated with thoracic length growth rate (p < 0.20). Sex was added to these variables to form the corresponding multivariate regression models.

**Fig. 3:**
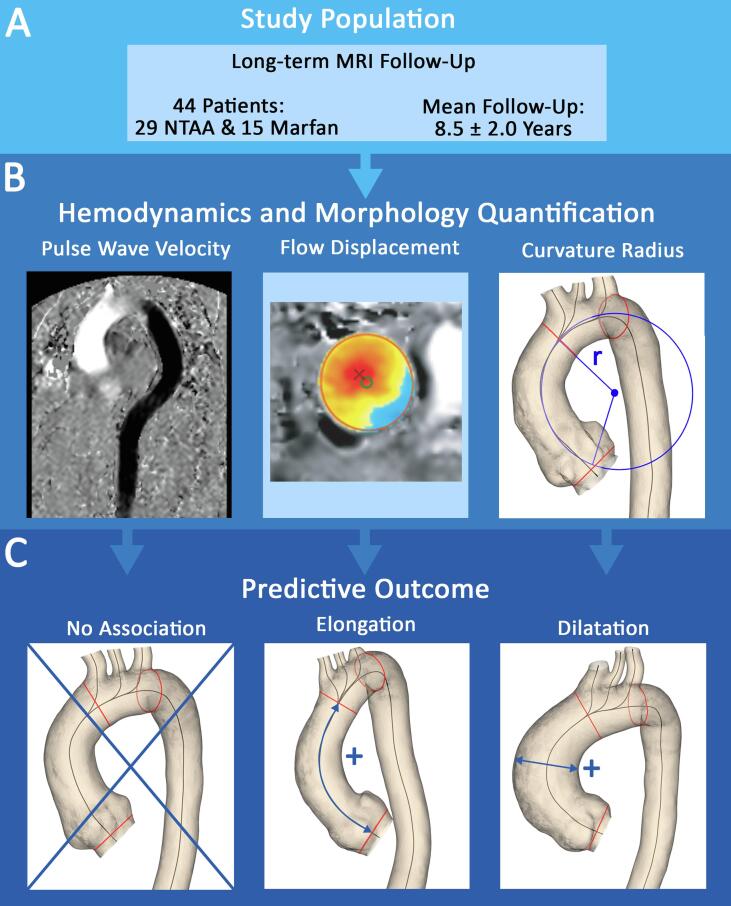
A: This study describes long-term MRI follow-up of NTAA and Marfan patients and investigates the predictive value of PWV, flow displacement and ascending aorta curvature radius on aortic growth. B: On the left, velocity-encoding image in feet-head direction illustrating in-plane PWV, in the middle an example of flow displacement and on the right the ascending aorta curvature radius. C: No association was found for PWV with aortic growth at follow-up, greater flow displacement predicted faster ascending aorta elongation and a smaller curvature radius predicted faster dilatation. *Abbreviations: MRI = magnetic resonance imaging, NTAA = non-syndromic thoracic aortic aneurysm PWV = pulse wave* velocity*.*

Regional PWV at baseline was not associated with regional aortic diameter or length growth during follow-up in univariate or multivariate regression in any of the segments (Table 3). We observed that the curvature of the ascending aorta was negatively associated with diameter growth of the ascending aorta at follow-up (β −0.018 mm [0.006], p = 0.01), meaning that for a smaller curvature the ascending aorta dilated faster compared to a larger ascending aorta curvature. This association remained significant after adjustment for age, sex, baseline diameter and heart rate (β −0.017 mm [0.007], p = 0.01; Table 4). Ascending aorta curvature radius was also negatively associated with elongation of the ascending aorta in univariate regression (β −0.037 mm [0.018], p = 0.04), however this was not significant after adjustment for age, sex, baseline length and history of hypertension and diabetes (β −0.026 mm [0.020], p = 0.21). Flow displacement at the ascending aorta was not associated with diameter growth in univariate or multivariate analysis (β −0.8 mm [1.2], p = 0.56; β −0.3 mm [1.4], p = 0.85, respectively). However, flow displacement was associated with ascending aorta elongation (β = 9.5 mm [3.2], p = 0.01), which remained significant after adjustment for age, sex, baseline length and history of hypertension and diabetes (β = 9.1 mm [3.3], p = 0.01). An overview of the main results is provided in Fig. 3.

**Table 3:** Univariate and multivariate regression of PWV versus diameter and length growth per aortic segment.

	Diameter growth / year (mm/y)		Length growth / year (mm/y)	
	*Beta [SE]*	*P-value*	*Beta [SE]*	*P-value*
Univariate				
PWV S1	−0.016 [0.010]	0.10	−0.028 [0.027]	0.30
PWV S2	0.010 [0.007]	0.17	0.028 [0.017]	0.12
PWV S3	0.013 [0.007]	0.07	0.023 [0.044]	0.61
PWV S4	−0.012 [0.022]	0.59	−0.037 [0.057]	0.53
PWV tot			−0.040 [0.077]	0.61

Multivariate*				
PWV S1	−0.011 [0.010]	0.28	−0.009 [0.031]	0.79
PWV S2	0.001 [0.007]	0.91	0.021 [0.018]	0.24
PWV S3	0.001 [0.007]	0.91	0.012 [0.053]	0.82
PWV S4	−0.017 [0.016]	0.29	−0.015 [0.077]	0.85
PWV tot			−0.098 [0.086]	0.27

* Diameter growth adjusted for age, sex, baseline diameter and heart rate; length growth adjusted for age, sex, baseline length and history of hypertension and diabetes. *Abbreviations: PWV = pulse wave velocity.*

**Table 4:** Univariate and multivariate regression of ascending aorta flow displacement and curvature versus diameter and length growth of the ascending aorta.

	S1 diameter growth / year (mm/y)		S1 length growth / year (mm/y)	
	*Beta [SE]*	*P-value*	*Beta [SE]*	*P-value*
Univariate				
Flow displacement	−0.8 [1.2]	0.56	9.5 [3.2]	0.01
Ascending aorta curvature radius	−0.018 [0.006]	0.01	−0.037 [0.018]	0.04

Multivariate*				
Flow displacement	−0.3 [1.4]	0.85	9.1 [3.3]	0.01
Ascending aorta curvature radius	−0.017 [0.007]	0.01	−0.026 [0.020]	0.21

* Diameter growth adjusted for age, sex, baseline diameter and heart rate; length growth adjusted for age, sex, baseline length and history of hypertension and diabetes.

Sensitivity analysis, in which we also included ascending aortic diameters based on clinically assessed TTE and CT images, showed that the association between ascending aorta curvature radius and ascending diameter growth remained significant (β −0.019 mm [0.006], p = 0.004) and that the associations for PWV and flow displacement remained non-significant (**Table S2).**

## 4 Discussion

In this study we investigated the impact of ascending aortic curvature radius, normalized FD and PWV on aortic growth in Marfan and NTAA patients and found that a smaller aortic curvature is associated with an increased ascending aorta dilatation rate and FD is associated with ascending aorta elongation at long-term follow-up (8.0 [7.3–10.7] years). Interestingly, PWV was not associated with aortic growth. Both aortic dilatation and elongation are associated with increased risk of dissection, markers that are able to predict either dilatation or elongation could aid in risk assessment of aortic pathology [3], [6].

### 4.1 Aortic curvature

The association of smaller ascending aorta curvature radius with accelerated aortic growth is consistent with the mathematical model showing that aortic curvature has more impact on the force of blood that is exerted on the aortic wall than other markers associated with accelerated aortic growth such as blood pressure, aortic diameter and patient size [9]. The alteration of blood flow patterns through a sharper curve has also been observed in a study investigating the angle between the heart and the aorta [19]. In that study it was observed that having a smaller heart-aorta angle was associated with increased wall shear stress particularly at the outer curvature of the proximal aorta in patients with ascending aorta dilatation. The impact of ascending aortic curvature on aortic dilation at follow-up has not been investigated previously. However, one recent cross-sectional study found that a smaller ascending aortic curvature angle may be a risk factor for developing a type-A dissection [20]. Accelerated aortic growth is also a risk factor for aortic dissection and is used in the guidelines to consider lower diameter thresholds for aortic intervention, therefore it may be useful to closely monitor patients with a small ascending aorta curvature radius [3].

### 4.2 Flow displacement

Aortic length is known to increase faster with age than aortic diameter, which is thought to be due to the fact that strain in the longitudinal direction is greater than in the circumferential direction [21]. Possibly, increased flow displacement in the mid-ascending aorta increases the longitudinal strain exerted on the ascending aorta, thereby increasing aortic length over time. This is the first study to report on the impact of FD on ascending aorta elongation. Thus far, aortic elongation has been underrecognized as morphologic parameter, however this changed in recent years as several studies have shown the increased risk of dissection associated with aortic elongation [5], [6]. These studies illustrate that the focus on aortic dilatation in the current guidelines does not sufficiently capture the three-dimensional structure and growth of the aorta. Given the increased risk associated with aortic elongation and the fact that over half of the patients with aortic dissection do not meet the current limit for elective surgical intervention, prediction of ascending aortic elongation could improve risk stratification in these patients [4].

In the current study with 8 years of follow-up, flow displacement did not predict diameter growth. A previous study has shown an association between FD and aortic growth at follow-up, however this has only been observed in bicuspid valve patients in a small sample size population (n = 25) [10]. In the current population there were no patients with a bicuspid valve. Bicuspid valve patients often have relatively high amounts of FD, also higher compared to our population, possibly explaining the absence of an association of FD with aortic dilatation in our study [22]. Also, in our study FD was assessed at the mid-ascending aorta, which may not capture aortic dilatation in the more proximal ascending aorta and aortic root.

### 4.3 Pulse wave velocity

In the current study we investigated the long-term predictive value of regional PWV assessed through in-plane velocity-encoded PWV on aortic growth. Previous studies in this population illustrated that normal regional PWV was associated with absence of dilated aorta and absence of growth at 2-year follow-up [7], [8]. In this long-term follow-up study, we did not find any associations between regional PWV and aortic growth. The prognostic value of aortic stiffness on aortic dilatation is still an ongoing debate, with studies showing conflicting results. Some studies have shown that arterial stiffness measures are associated with aortic growth rate, while others have found the exact opposite and showed that arterial stiffness is associated with slower aortic growth [23], [24]. In accordance with our study, one other study has also reported no impact of aortic stiffness on aortic growth [25]. These differences may be partially explained by the different techniques and imaging modalities used to determine aortic stiffness, which range from MRI distensibility to TTE elastic modulus and applanation tonometry PWV. In the current study we used in-plane velocity-encoded PWV with high temporal resolution, which has shown higher agreement with invasive aortic pressure measurements (the gold standard for PWV) as compared to through-plane PWV [11]. The lack of an association may indicate that PWV is a less important marker for long term outcome than previously thought and perhaps we should focus more on parameters like aortic curvature radius and flow displacement. Future (meta)analysis of previous studies could provide more insight into the association between arterial stiffness and aortic growth rate.

## 5 Limitations

There are limitations that need consideration. The sample size is relatively small, however this is inherent to the long-term follow-up of complex baseline MRI measures, which at that time were state of the art and used only in a small population. The diameter assessed at baseline used contrast-enhanced MRA images and the diameter at follow-up was assessed using non-contrast enhanced Dixon images, although both have shown diagnostic accuracy in assessment of aortic dilatation [26]. In this study we used 2D through-plane velocity-encoding for assessment of flow displacement. Both 2D and 4D-flow can be used for the assessment of FD, however, the positioning of the 2D phase-contrast MRI acquisition on the ascending aorta may not be at the position with the most prominent flow displacement in the flow pattern [13]. Still, a previous study that assessed FD compared 2D and 4D-flow and found good agreement between both [27]. Finally, since no 4D flow MRI was performed at baseline, no information on wall shear stress could be obtained.

## 6 Conclusion

In Marfan and non-syndromic thoracic aortic aneurysm patients, ascending aorta curvature radius and flow displacement are associated with accelerated aortic growth rate at long-term follow-up. These markers may aid in the risk stratification of ascending aorta elongation and aneurysm formation.

## Declaration of Competing Interest

The authors declare that they have no known competing financial interests or personal relationships that could have appeared to influence the work reported in this paper.
