# Supplementary material for: Ascending aorta curvature and flow displacement are associated with accelerated aortic growth at long-term follow-up: A MRI study in Marfan and thoracic aortic aneurysm patients
Source: Int J Cardiol Heart Vasc. 2021 Dec 13;38:100926. doi: 10.1016/j.ijcha.2021.100926 (PMC8683588; doi:10.1016/j.ijcha.2021.100926)
Supplement: Supplementary data 1 [file mmc1.docx]

**Table S1:** baseline characteristics including patients with CT and TTE follow-up

|  | **NTAA**  *n = 35* | **Marfan**  *n = 19* | **Total**  *n = 54* |
| --- | --- | --- | --- |
| Age (years) | 56.0 ± 13.0 | 33.9 ± 13.2 | 48.2 ± 16.8 |
| Sex (% male) | 25 (71.4 %) | 9 (47.4 %) | 34 (63.0 %) |
| Height (cm) | 179.3 ± 10.1 | 186.6 ± 9.4 | 181.8 ± 10.4 |
| Weight (kg) | 85.9 ± 14.5 | 86.5 ± 21.2 | 86.1 ± 17.0 |
| BSA (m^2^) | 2.0 ± 0.2 | 2.1 ± 0.3 | 2.1 ± 0.2 |
| SBP (mmHg) | 135.9 ± 20.3 | 123.6 ± 11.2 | 131.2 ± 18.3 |
| DBP (mmHg) | 80.7 ± 10.7 | 72.8 ± 8.8 | 77.7 ± 10.6 |
| MAP (mmHg) | 99.1 ± 12.0 | 89.7 ± 8.5 | 95.5 ± 11.7 |
| Heart rate (beats/min) | 66.2 ± 10.5 | 66.5 ± 10.1 | 66.3 ± 10.3 |
| History of   - Hypertension (%) - Dyslipidaemia (%) - Diabetes (%) - Smoking (%) - Bicuspid aortic valve (%) | 24 (68.6 %)  15 (42.9 %)  1 (2.9 %)  7 (20.0 %)  0 (0.0 %) | 2 (10.5 %)  1 (5.3 %)  0 (0 %)  3 (15.8 %)  0 (0.0 %) | 26 (48.1 %)  16 (29.6 %)  1 (1.9 %)  10 (18.5 %)  0 (0.0 %) |
| Use of   - AT2 / ACE inhibitor (%) - Betablocker (%) - Statin (%) | 19 (54.3 %)  16 (45.7 %)  13 (37.1 %) | 5 (26.3 %)  15 (78.9 %)  0 (0 %) | 24 (44.4 %)  31 (57.4 %)  13 (24.1 %) |
| Left ventricular ejection fraction | 61.4 ± 5.1 | 60.5 ± 6.7 | 61.1 ± 5.7 |
| Baseline diameter S1 (mm) | 42.3 ± 4.1 | 38.6 ± 3.7 | 40.9 ± 4.4 |
| Baseline diameter S2 (mm) | 34.6 ± 4.1 | 26.9 ± 2.6 | 31.9 ± 5.2 |
| Baseline diameter S3 (mm) | 28.2 ± 2.6 | 24.5 ± 3.5 | 26.9 ± 3.4 |
| Baseline diameter S4 (mm) | 21.8 ± 2.4 | 18.7 ± 2.9 | 20.7 ± 3.0 |
|  |  |  |  |
| Baseline length S1 (mm) | 95.3 ± 13.0 | 82.8 ± 11.5 | 90.9 ± 13.7 |
| Baseline length S2 (mm) | 38.7 ± 8.1 | 32.5 ± 4.3 | 36.5 ± 7.6 |
| Baseline length S3 (mm) | 272.7 ± 24.2 | 251.3 ± 24.5 | 265.1 ± 26.2 |
| Baseline length S4 (mm) | 101.4 ± 10.8 | 104.6 ± 12.6 | 102.6 ± 11.5 |
|  |  |  |  |
| PWV S1 (m/s) | 6.8 ± 3.6 | 6.4 ± 5.9 | 6.6 ± 4.5 |
| PWV S2 (m/s) | 8.1 ± 5.6 | 6.2 ± 5.1 | 7.4 ± 5.5 |
| PWV S3 (m/s) | 8.2 ± 3.7 | 6.6 ± 4.4 | 7.7 ± 4.0 |
| PWV S4 (m/s) | 7.9 ± 4.0 | 7.9 ± 3.8 | 7.9 ± 3.9 |
|  |  |  |  |
| Flow displacement | 0.05 ± 0.04 | 0.04 ± 0.01 | 0.05 ± 0.03 |
| Ascending aorta curvature radius (mm) | 46.5 ± 7.2 | 44.3 ± 5.9 | 45.7 ± 6.8 |
| *Data are shown as n (%), mean ± SD or Abbreviations: BSA = body surface area, CT = computed tomography, DBP = diastolic blood pressure, MAP = mean arterial pressure, NTAA = non-syndromic thoracic aortic aneurysm, PWV = pulse wave velocity, SBP = systolic blood pressure, TTE = transthoracic echocardiography.* | | | |

**Table S2:** Sensitivity analysis of PWV, flow displacement and ascending aorta curvature radius with diameter growth including follow-up with transthoracic echocardiography and CT.

|  | S1 diameter growth / year (mm/y) | |
| --- | --- | --- |
|  | *Beta [SE]* | *P-value* |
| Univariate |  |  |
| PWV S1 | -0.013 [0.010] | 0.18 |
| Flow displacement | -0.949 [1.228] | 0.44 |
| Ascending aorta curvature radius | -0.018 [0.006] | 0.002 |
|  |  |  |
| Multivariate* |  |  |
| PWV S1 | -0.012 [0.010] | 0.25 |
| Flow displacement | -0.173 [1.330] | 0.90 |
| Ascending aorta curvature radius | -0.019 [0.006] | 0.004 |

* Adjusted for age, sex, baseline diameter and heart rate. *Abbreviations: PWV = pulse wave velocity.*
